# Tennis player actions dataset for human pose estimation

**DOI:** 10.1016/j.dib.2024.110665

**Published:** 2024-06-22

**Authors:** Chun-Yi Wang, Kalin Guanlun Lai, Hsu-Chun Huang, Wei-Ting Lin

**Affiliations:** a Office of Physical Education, National Taichung University of Science and Technology, Taichung, Taiwan; b Department of Computer Science & Information Engineering, National Kaohsiung University of Science and Technology, Kaohsiung, Taiwan; c Department of Mechanical Engineering, National Kaohsiung University of Science and Technology, Kaohsiung, Taiwan; d Physical Education Office, National Kaohsiung University of Science and Technology, Kaohsiung, Taiwan

**Keywords:** Human posture recognition, Pose estimation, Keypoint detection, Tennis action, COCO, Sports Technology

## Abstract

Tennis is a popular sport, and integrating modern technological advancements can greatly enhance player training. Human pose estimation has seen substantial developments recently, driven by progress in deep learning. The dataset described in this paper was compiled from videos of researchers’ friend playing tennis. These videos were retrieved frame by frame to categorize various tennis movements, and human skeleton joints were annotated using COCO-Annotator to generate labelled JSON files. By combining these JSON files with the classified image set, we constructed the dataset for this paper. This dataset enables the training and validation of four tennis postures, forehand shot, backhand shot, ready position, and serves, using deep learning models (such as OpenPose). The researchers believe that this dataset will be a valuable asset to the tennis community and human pose estimation field, fostering innovation and excellence in the sport.

Specifications Table

**Table:** 

Subject	Computer Science / Computer Vision and Pattern Recognition; Data Science / Applied Machine Learning
Specific subject area	Human Posture Recognition; Action Recognition; Pose Estimation; Keypoint Detection
Data format	Filtered
Type of data	.jpeg file (the images from video's frame) .json file (COCO-format)
Data collection	The dataset comprises 4 different actions in tennis, each action has 500 images and a COCO-format JSON files.The actions in this dataset, and the action categories name in COCO-format is in brackets: 1. backhand shot (backhand) 2. forehand shot (forehand) 3. ready position (ready_position) 4. serve (serve) We organize two main directories: annotations and images. • annotations: the JSON files of the actions (COCO-format) ▪ We use COCO-Annotator to annotating and categorizing human actions. • images: the images of the actions (according four actions classify to four folders) ▪ The images in the dataset were extracted frame by frame from videos that were self-recorded, and manually classified according to different tennis actions.
Data source location	Taipei Tennis Center, in Taipei City, Taiwan.
Data accessibility	Repository name: Tennis Player Action Dataset for Human Pose Estimation Data identification number: 10.17632/nv3rpsxhhkt Direct URL to data: https://data.mendeley.com/datasets/nv3rpsxhhk

## 1 Value of the Data

• This dataset has significantly contributed to sports technology by integrating computer vision techniques to further the advancement of sports tech.
• Employing the widely used COCO-format and annotates human skeletal joints (key points), facilitating easy access and training for users.
• The dataset is meticulously curated to capture the nuances of tennis movements, providing detailed annotations for a variety of actions such as serves, volleys, and groundstrokes. This allows for the development of precise pose estimation models that are highly effective in analyzing and enhancing tennis performance.
• If needed, users can also utilize this dataset for other applications, such as tracking tennis balls, by labeling and training it on their own.

## 2 Background

Datasets related to sports provide valuable data for a wide range of research fields, including policy-making, education, public health, and sports science. Traditionally, these datasets mainly contain raw statistics on athletes' physical conditions, outputs from various modeling efforts, or data collected through software tools, all of which contribute significantly to the advancement of these fields [[1], [2], [3], [4]]. As technology progresses, computer vision has emerged as a critical area of research, especially in human pose estimation. This domain has witnessed the development of specific datasets to support such research efforts [[5], [6]]. Consequently, several datasets within the field of sports science are now specifically designed for training human pose estimation models. For instance, LDCNet focuses on flexible human pose estimation by leveraging limb direction cues, highlighting its application in industrial behavioral biometrics systems [7]. Another example is ARHPE, which employs asymmetric relation-aware representation learning to enhance head pose estimation, a crucial aspect in industrial human-computer interaction [8]. Additionally, MFDNet advances the field by integrating collaborative pose perception with matrix Fisher distribution for precise head pose estimation [9]. These datasets are integral in supporting the development and refinement of human pose estimation models, facilitating advancements in sports science and other related fields.

Traditional practices for creating datasets have predominantly relied on computer vision techniques. However, with the advent and evolution of deep learning, the need for raw image files and annotation data has become paramount. The COCO format has emerged as a standard for annotation data in recent years [8]. Our dataset also utilizes the COCO format, facilitating its use for training and validation in deep learning models such as OpenPose [9] and MediaPipe [10]. This dataset comprises a comprehensive collection of annotated tennis action images, designed to train models capable of recognizing specific postures within tennis matches. Moreover, users can customize the dataset for other applications, such as tracking tennis balls, by adding their own labels and training data. The primary objective of this dataset is to promote the advancement of computer vision applications in tennis-related fields. In recent years, several significant advancements have been made in the field of computer vision and deep learning. For example, OpenPose represents a notable improvement over traditional pose estimation models by providing a multi-person pose detection framework, which enhances the accuracy and applicability of pose recognition [9]. Similarly, MediaPipe has extended these capabilities by integrating real-time pose estimation with high efficiency, making it a preferred tool for a wide range of applications beyond sports [10]. The progression from basic pose estimation models to more sophisticated frameworks illustrates the rapid development and increasing precision of computer vision technologies, which our dataset aims to support.

## 3 Data Description

**Fig. 1:**
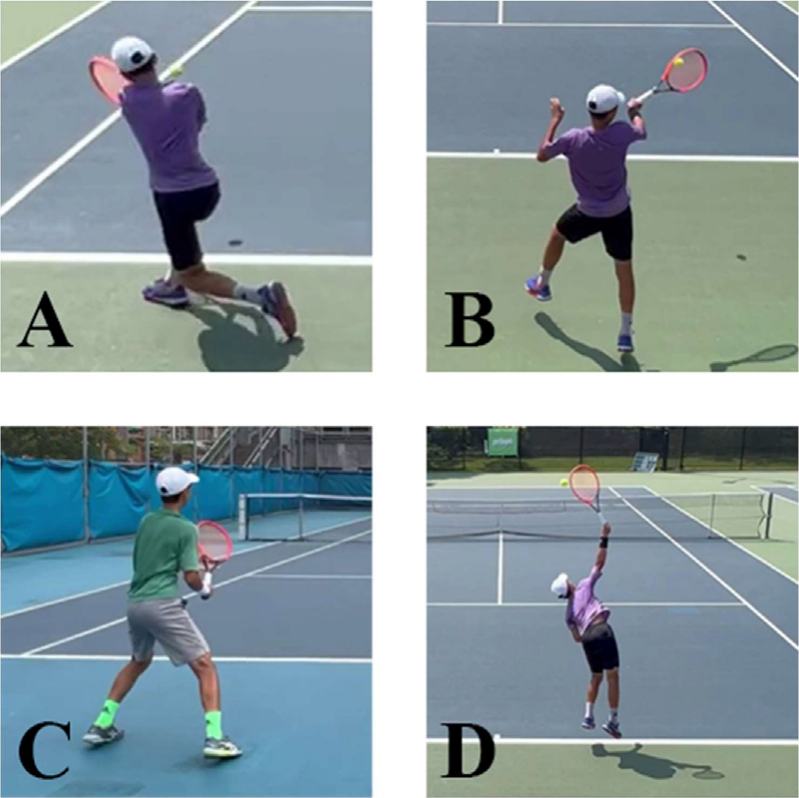
Common tennis postures. (A) Backhand stroke, (B) Forehand stroke, (C) Ready Position, (D) Serve.

This dataset is designed for human pose estimation applications within tennis, featuring commonly observed tennis postures including forehand stroke, backhand stroke, ready position, and serve, as shown in Fig. 1.

**Fig. 2:**
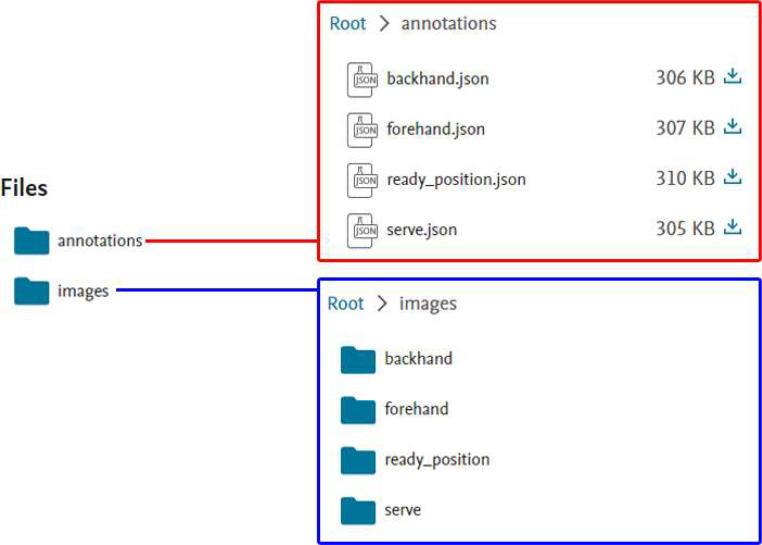
Dataset files on Mendeley Data.

This dataset contains two parts: 1. images from the frame of the video of the players’ action, and 2. the action annotation JSON files (COCO-format). Part 1 have 2,000 images, part 2 have 4 files, and it on Mendeley Data shown in Fig. 2 (size on disk is about 508 MB (533,372,928 bytes)).

**Fig. 3:**
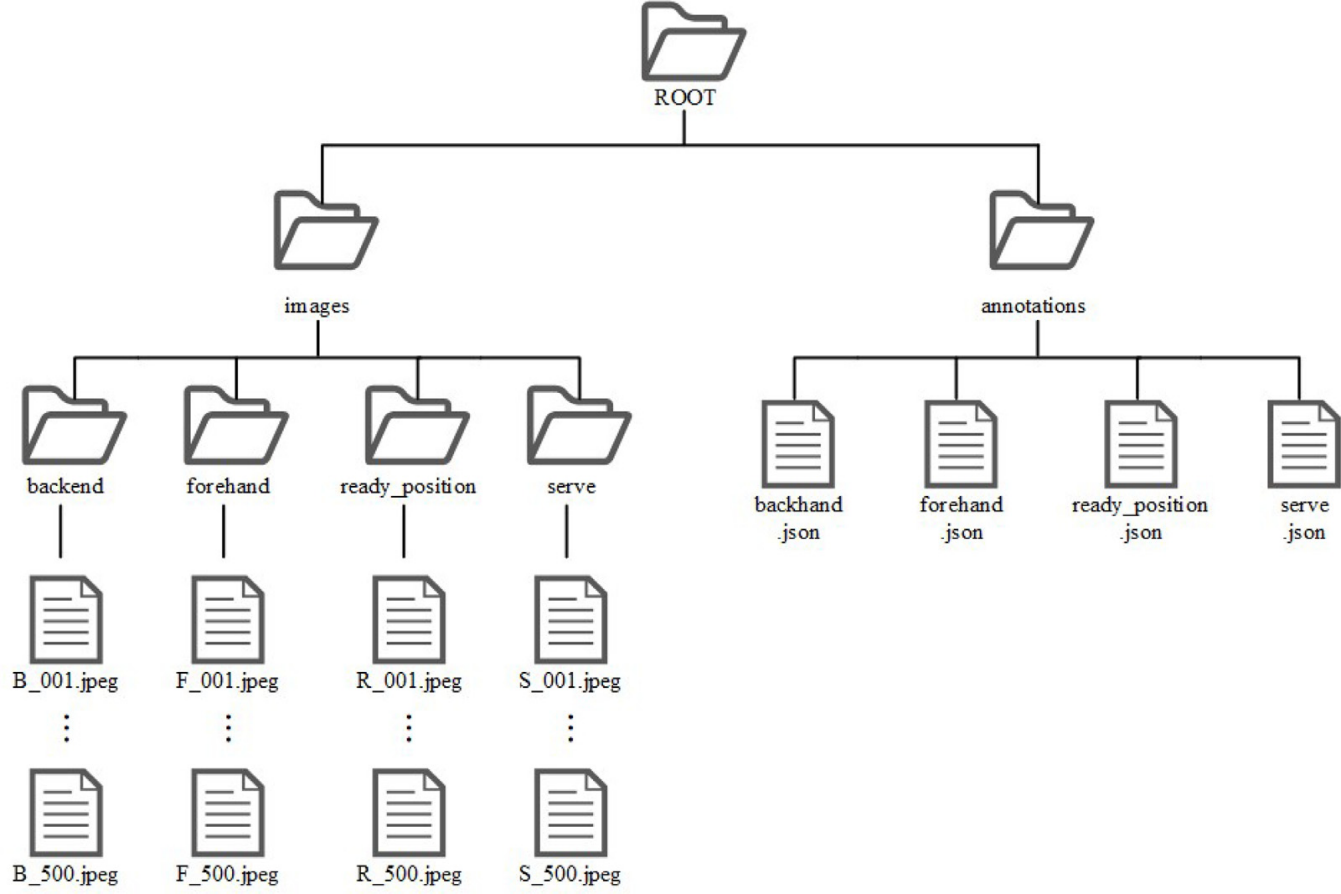
Folder structure.

The researchers organized two parts as two main directories. One is images, it divided into four subfolders by posture in ``images'' folder. The files in the subfolders are named by researchers, following a specific convention. Researchers extract the first letter of the parent folders and assign sequential numbering. For instance, the name of the images within ``images/backhand'' folder have prefix ``B_'', and followed by a numerical sequence (e.g., B_001, B_002, …, B_500). The Other is annotation JSON files, it has four files and named by four postures. Folder structure is shown in Fig. 3.

## 4 Image Information

The dataset described in this article contains 500 images for each posture, total have 2000 images. Before classified to four specific actions, the researchers first recorded videos of themselves playing tennis, then analyzed these videos’ frame by frame to classify the frames’ image into the specified actions. These videos were captured using a smartphone with a resolution of 720P, with dimensions of 1280 pixels width and 720 pixels height, and a frame rate of 30 fps, so the images’ resolution also is 1280 × 720. The data collection outline is shown in Table 1.

**Table 1:** Brief description about the data collection.

No.	Particulars	Description
1	Data type	4 tennis postures: (1) forehand stroke (2) backhand stroke (3) ready position (4) serve
2	Original data format	Video file using H.264/MPEG-4 AVC codec (.mp4) Resolution: 720P (1280 × 720 pixels) Frame Rate: 30 fps
3	Filtered data format	JPEG image file (.jpeg) Resolution: 720P (1280 × 720 pixels)
4	Period and Date	January-December 2023
5	Participants	Member of the World Junior Team Championships (Lin,yu-min. Taiwan)
6	Location	Taipei Tennis Center

*Source:* Author's own organization.

## 5 Annotation File Information

**Fig. 4:**
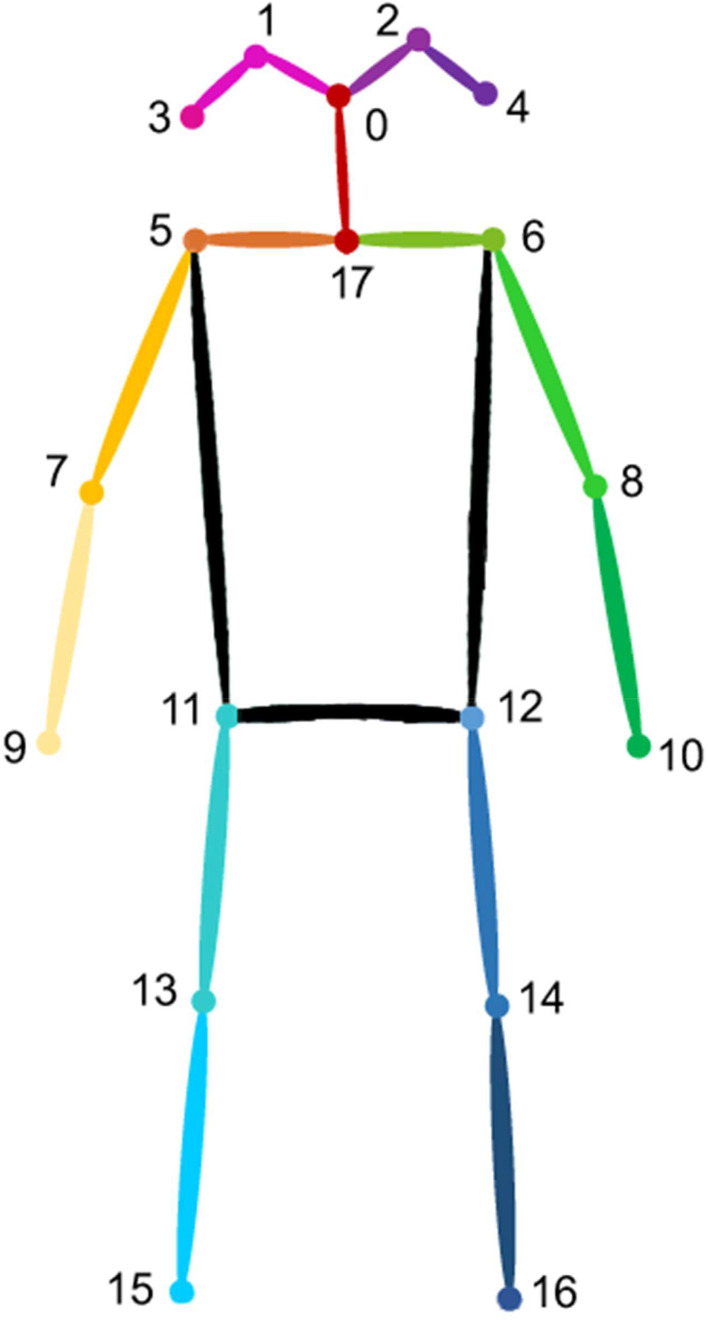
Human skeleton joints in the dataset.

The researchers utilize the extracted images to annotate human skeletal joints and classify the postures. For supplying common deep learning model of human pose estimation to train, the researchers use COCO-Annotator [11] as annotation tool, annotated joints are illustrated in Fig. 4 and the joints number and name pairs are shown in Table 2.

**Table 2:** Skeleton joint number to name pairs in Fig. 4.

Joint's No.	Joint's Name
0	nose
1	left_eye
2	right_eye
3	left_ear
4	right_ear
5	left_shoulder
6	right_shoulder
7	left_elbow
8	right_elbow
9	left_wrist
10	right_wrist
11	left_hip
12	right_hip
13	left_knee
14	right_knee
15	left_ankle
16	right_ankle
17	neck

*Source:* Author's own organization.

**Fig. 5:**
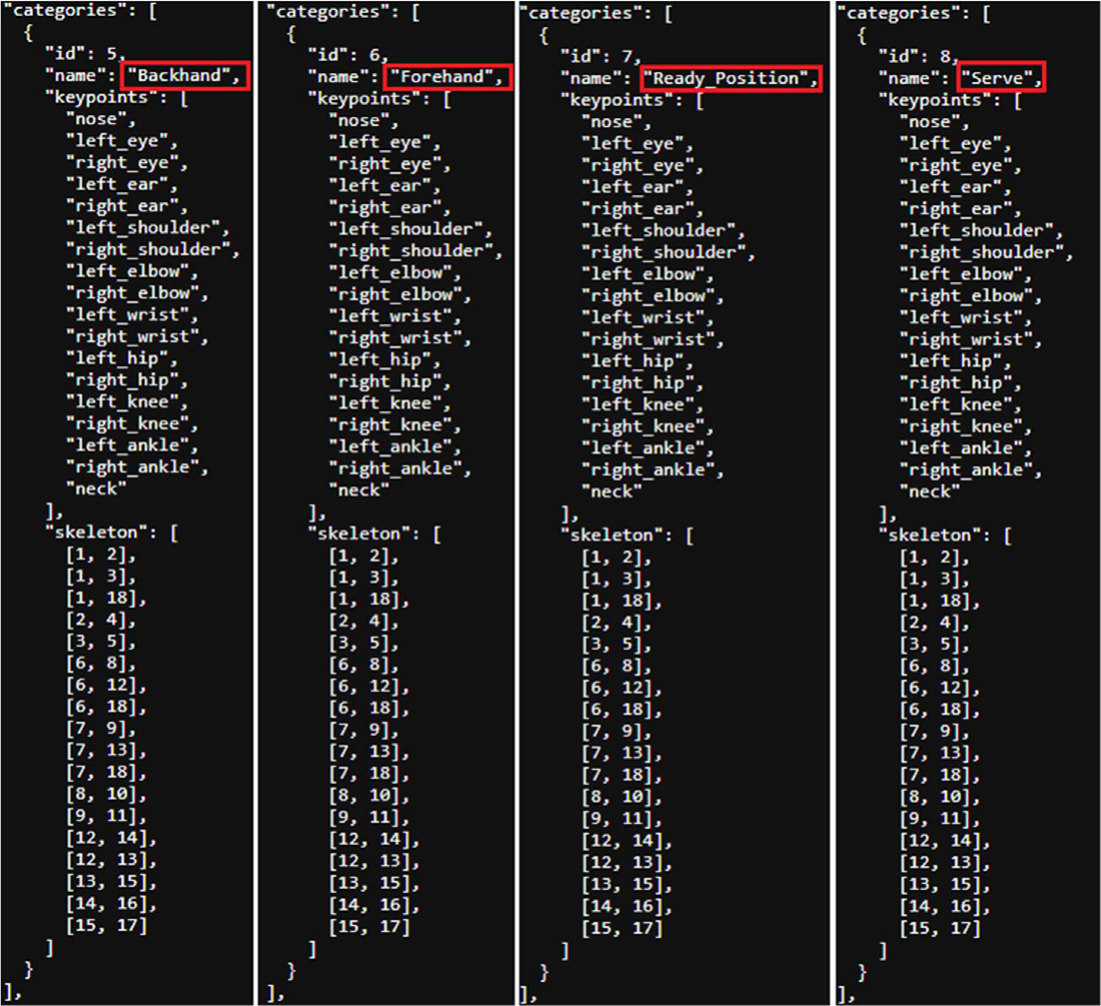
The instance of the categories’ part in JSON file (for 4 postures).

**Fig. 6:**
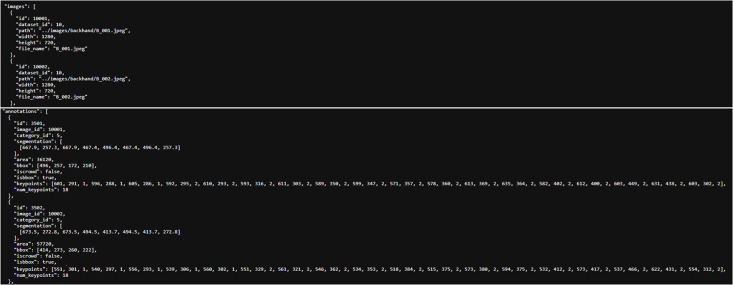
The instance of the part of image and annotation in JSON file (from 2 backhand images).

Because the researchers use COCO-Annotator, so the generated JSON files must be COCO format. The JSON file's format in this dataset is shown in Table 3. The instances of categories’ name, keypoints, and skeleton in JSON file is represent to Fig. 5, the name of category in the red box is the posture's name, the category information is set by researchers using COCO-Annotator. The instance of image and annotation in the JSON file is represent to Fig. 6.

**Table 3:** JSON file's format of this dataset.

{ ``images'': [ { ``id'': (image ID, same as the id in part of ``annotations''), ``dataset_id'': (dataset ID), ``path'': (image file path), ``width'': (image width), ``height'': (image height), ``file_name'': (image file name) }, … ], ``categories'': [ { ``id'': (category ID), ``name'': (category name, same as the id in part of ``annotations''), ``keypoints'': (keypoints list), ``skeleton'': (skeleton (connected keypoints) list) } ], ``annotations'': [ { ``id'': (annotation ID), ``image_id'': (image ID, same as the id in part of "images"), ``category_id'': (category ID, same as the id in part of ``category''), ``segmentation'': (polygon list), ``area'': (the area of the target box. unit: pixel), ``bbox'': (the coordinates list of the target box’ each corners), ``iscrowd'': (whether the image is a crowd), ``isbbox'': (whether the image has target box), ``keypoints'': (list of keypoints coordinates on the image), ``num_keypoints'': (number of keypoints on the image) }, … ] }

*Source:* Author's own organization.

## 6 Experimental Design, Materials and Methods

**Fig. 7:**
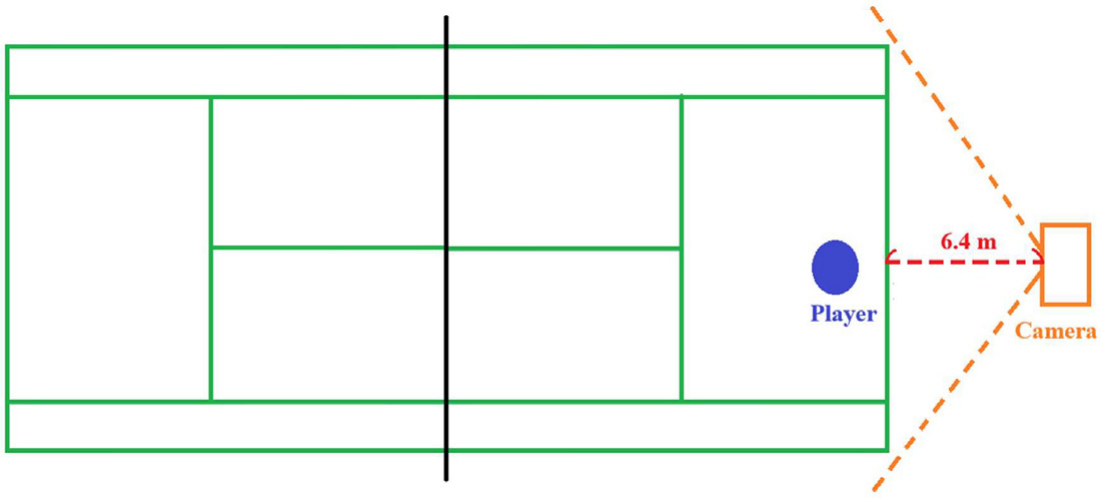
Location of camera setup in the experimental field.

The camera is positioned at the rear of the tennis court, capturing the player from behind (aligned with the player's facing direction). The camera is positioned approximately 6.4 meters from the court's baseline. The height, while not fixed or recorded, is considered by researchers to be inconsequential for the purpose of analyzing tennis movements. The location of the camera setup is shown in Fig. 7.

**Fig. 8:**
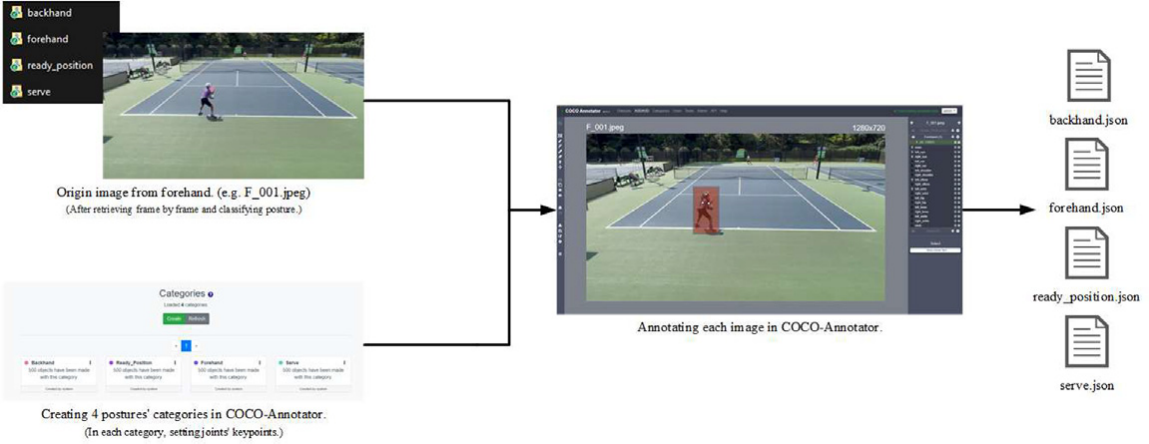
The process flow of annotating using COCO-Annotator.

Before playing tennis, we set up an experimental recording environment like above. Once the setup is complete, we began recording the video. Subsequently, we retrieve the video frame by frame, classifying each frame according to the specific tennis action it captures to compile an image dataset. Then the researchers annotated target human's skeleton joint for each image in these images using COCO-Annotator and generate labeled JSON files to construct the dataset for this article. The process flow is illustrated in Fig. 8.

## Limitations

When creating this dataset, the camera setup was not at a fixed distance; it was simply positioned behind the player to record the various strokes. The dataset currently includes only four actions: forehand shot, backhand shot, ready position, and serve. While these encompass most of the essential tennis movements, some minor actions might still be missing. In the future, we aim to expand the collection to include a broader range of movements and to augment the dataset with additional images.

## Ethics Statement

After the performance of the experiment, we blurred all unrelated people in images (the people of the opposite field). And the participant (the person back on the camera) in images is the authors’ friend, he provided some data related to physical status and habits of individual, and they read and signed an informed consent form, conserved at Physical Education Office at ``National Kaohsiung University of Science and Technology'' (the correspondent's office). We follow Research ethics guidelines in everything we do, and have obtained a certificate from the local Center for Taiwan Academic Research Ethics Education, certificate number: P107259575-1.

## CRediT authorship contribution statement

**Chun-Yi Wang:** Conceptualization, Data curation, Methodology. **Kalin Guanlun Lai:** Software, Writing – original draft. **Hsu-Chun Huang:** Validation, Writing – review & editing. **Wei-Ting Lin:** Supervision, Validation, Writing – review & editing.

## Data Availability

Tennis Player Actions Dataset for Human Pose Estimation (Original data) (Mendeley Data).
